# Distribution, treatment outcome and genetic diversity of *Leishmania* species in military personnel from Colombia with cutaneous leishmaniasis

**DOI:** 10.1186/s12879-020-05529-y

**Published:** 2020-12-09

**Authors:** Camilo A. Correa-Cárdenas, Julie Pérez, Luz H. Patino, Juan David Ramírez, Maria Clara Duque, Yanira Romero, Omar Cantillo-Barraza, Omaira Rodríguez, Maria Teresa Alvarado, Claudia Cruz, Claudia Méndez

**Affiliations:** 1 Grupo de Investigación en Enfermedades Tropicales del Ejército (GINETEJ), Laboratorio de Referencia e Investigación, Dirección de Sanidad Ejército, Bogotá, Colombia; 2 Grupo de Investigaciones Microbiológicas – UR (GIMUR), Departamento de Biología, Facultad de Ciencias Naturales, Universidad del Rosario, Bogotá, Colombia

**Keywords:** *Leishmania* spp., Cutaneous leishmaniasis, *HSP70*, Phylogenetics, Genetic diversity, The Colombian National Army

## Abstract

**Background:**

Leishmaniasis is one of the most important infectious diseases affecting the Colombian National Army due to the high number of reported cases and exposure throughout military operations in endemic areas. The main aim of this study was to estimate the geographical distribution along with the genetic diversity and treatment outcome of *Leishmania* species in Colombian military personnel.

**Methods:**

Skin lesion samples by smear and aspirate were collected in 136 patients having parasitological cutaneous leishmaniasis (CL) diagnosis. DNA was extracted, the nuclear marker heat shock protein 70 (*HSP70*) was amplified by PCR and sequenced. *Leishmania* species were identified by BLASTn. The geo-spatial distribution of the identified parasites was determined according to the possible site of infection. Gene tree was constructed by maximum likelihood (ML), diversity indices (*π, h*) were estimated and haplotype network was constructed under the Templeton-Crandall-Sing algorithm in order to determine the geographic relationships of the genetic variants of *Leishmania* species circulating in Colombian military population.

**Results:**

The species were identified in 77.94% of the samples, with a predominance of *L. braziliensis* (65.09%), followed by *L. panamensis* (31.13%), *L. naiffi* by the first time reported in Colombia in two patients (1.89%) as well as *L. lindenbergi* in a single patient (0.945%) with possible infection in the municipality of Miraflores, Guaviare and *L. infantum* in a single patient (0.945%) notified with CL in the municipality of Tumaco, Nariño. The phylogenetic analysis was consistent according to bootstrap, showing four strongly differentiated clades.

**Conclusions:**

The geo-spatial distribution suggested that *L. braziliensis* has a greater abundance, while *L. panamensis* has a greater dispersion. The phylogenetic relationships of *Leishmania* species in Colombian military personnel was estimated with the confirmation of two new species circulating without prior report in the country and a species with no background for CL in the Colombian army. A substantial genetic diversity of *Leishmania braziliensis* was defined. This study contributes through the understanding of the molecular epidemiology to the CL transmission in Colombia.

**Supplementary Information:**

The online version contains supplementary material available at 10.1186/s12879-020-05529-y.

## Background

The *Leishmania* parasites are the etiological agents of leishmaniasis, a tropical vector-borne disease (VBD) transmitted to humans through a bite of infected hematophagous female insects of the Psychodidae subfamily [1]. There are approximately 20 *Leishmania* species that can generate different clinical manifestations such as cutaneous leishmaniasis (CL), mucocutaneous leishmaniasis (MCL) or visceral leishmaniasis (VL) [2, 3]. Seventeen countries of the Americas reported to the Pan-American Health Organization (PAHO) and the World Health Organization (WHO) a total of 989.096 CL and MCL cases from 2001 to 2018 with a decreasing trend with some exceptions. Colombia is the 2nd (13.82%) after Brazil (35.69%) with the highest number of CL and MCL cases as well as the eighth (incidence: 26.17) with the highest disease incidence in the continent according to 2018 data [4]. A total of 6426 cases of leishmaniasis were reported during 2018 in Colombia in its three clinical forms corresponding to 98.3% (6319) CL, 1.4% (90) MCL, and 0.3% (17) VL cases [5]. CL is currently the infectious disease that most affects the Colombian National Army, where 41.774 cases have been reported between 2008 and 2019, followed by 575 cases of MCL in active military personnel, according to data from the National Surveillance System in Public Health (SIVIGILA) and Operational Health department of the Army Health Directorate [6].

Molecular markers such as the heat shock protein gene (*HSP70*) can be sequenced to identify by barcoding the *Leishmania* species [7, 8]. Studies conducted in Colombia have shown that in civil population a predominance of *L. braziliensis* (86.42%) exists, followed by *L. panamensis* (11.11%) by using of PCR-RFLP of the nuclear marker *HSP70* [9], while others found a predominance of *L. panamensis* (61.3%), followed by *L. braziliensis* (23.1%) by sequencing of the mitochondrial marker *Cytb* [7] as well as a predominance of *L. panamensis* (56.3%) followed by *L. braziliensis* (36.8%), *L guyanensis* and *L. amazonensis* by PCR-RFLP and sequencing of mini-exon and *HSP70* markers [10]. Regarding the military population, it has been reported to date the highest predominance of *L. braziliensis* (95.4%), followed by *L. guyanensis* (2.3%) throughout PCR-RFLP [11], compared to a predominance of *L. braziliensis* (61.1%), followed by *L. panamensis* (33.5%) using *Cytb* and *HSP70* markers sequencing [8].

Colombia is one of the countries with the greatest richness of human pathogenic *Leishmania* species (nine species) [7, 12, 13], moving the CL prevalence from sylvatic to domestic cycles in most of the country [14], and the military activities in sylvatic areas are considered one of the factors related to changes in the dynamics of disease transmission [8]. Due to variations in the relative abundances of *Leishmania* species range in different studies, it is worth conducting studies in order to understand the factors that determine the molecular epidemiology of *Leishmania* parasites in relation to the geographical origins [7, 8]. Therefore, the aim of this study was to determine the geographical distribution and genetic diversity of *Leishmania* species in military personnel with CL.

## Methods

### Sampling size

A sample size of 136 patients from leishmaniasis centers in Bonza, Boyacá (89 patients) and health battalion (BASAN) in Bogotá (47 patients) was estimated in Epi Info v7.2.2.6 (https://www.cdc.gov/epiinfo/index.html), with 95% CI and an expected sampling error of 5% based on the average number of patients treated for one year in these military establishments. Two groups of patients were defined: first group made up of those who received first-line treatment with meglumine antimoniate and the second group for those who had presented therapeutic failure with meglumine antimoniate and initiated second-line treatment with pentamidine isothionate. The study had as inclusion criteria patients who participated voluntarily from male military population, older than 18 years old, with a parasitological diagnosis of CL and where lesion size larger than 1 cm in diameter and without clinical evidence of bacterial or fungal infection at the beginning of the study. As an exclusion criterion was considered patients with facial, genitals lesions and / or presenting comorbidities as pathologies where treatment is contraindicated.

### Sampling, storing and medical resolution

In the case of smear samples to identify *Leishmania* species by barcoding, hand ischemia was performed using thumb-index fingers, scraped off the base and center of the ulcer with a sterile lancet [15]. Smear samples obtained were deposited in a screw cap vial with 250 μL of sterile saline solution. It was subsequently stored at − 70 °C until processing.

The clinical-epidemiological record was filled out by all of the patients with variables such as age, number of lesions, disease evolution, internal and external areas of lesions and municipality of possible infection. Once the patients were diagnosed, they were treated with the first line or second line treatment.

### DNA extraction, PCR, purification and sequencing

DNA extraction was carried out with the Invisorb® Spin Universal commercial kit - Stratec molecular (Berlin, Germany) according to the manufacturer’s indications. HSP70F (5 ‘AGG TGA AGG CGA CGA ACG 3’) and HSP70R (5 ‘CGC TTG TCC ATC TTT GCG TC 3’) primers were used to amplify a 337 bp partial region of *HSP70* [16]. PCR mix for *HSP70* consisted Master Mix Green Promega 1X PCR (Madison-WI, USA), 5 μM of each primer and 3 μL of DNA for a final volume of 15 μL. The thermal profile consisted of a pre-denaturation at 94 °C for 5 min, followed by 40 cycles with denaturation at 94 °C for 1 min, annealing at 58 °C for 1 min and elongation at 72 °C for 1 min. Then a final extension at 72 °C for 10 min [16]. All PCR products were verified by 1% agarose gel, stained with Gel Red™ 1000X and run at 80 V for 40 min in a horizontal electrophoresis system with TAE 1X. Finally, PCR products were subsequently purified by ExoSAP-IT and sequenced by the terminal dideoxy method using Big Dye chemistry v 3.1 within an AB3730xI automatic sequencer in Macrogen Korea.

### Sequence editing and species identification

*HSP70* sequences were edited manually using Geneious Pro 4.8.1 software (http://www.geneious.com) [17]. Sequence alignment was performed under the MUSCLE algorithm [18] and identity of sequences was confirmed with BLASTn in NCBI (http://blast.ncbi.nlm.nih.gov), where the *Leishmania* species was identified by the similarity between study sequences and others deposited in GenBank. All haplotypes were identified using MacClade software v 4.0.8 [19, 20]. A geo-referenced database was constructed to address the spatial distribution of identified *Leishmania* species. Sample data belong to the possible site of infection, as reported by the patient. Distribution and frequency of the parasite species was mapped by using ArcGIS10.3 [21].

### Phylogenetic analysis

Sequences were obtained from GenBank for the phylogenetic analyses. The following sequences were downloaded for the phylogenetic reconstruction: *Leishmania major* (FN395023 and HF586344), *L. aethiopica* (FN395018 and FN395019), *L. tropica* (FN395025 and FN395026), *L donovani* (FN395027 and HF586385), *L. mexicana* (EU599091, HF586401 and FN395038), *L. amazonensis* (HF586353, LN907831 and MF344846), *L. lainsoni* (FN395047, FN395048 and LN907839), *L. peruviana* (EU599089, FN395044 and FR872765), *L. guyanensis* (FN395052, LN907836 and MG029128) and *L. shawi* (GU071177 and MG029127) [22–27]. Additionally, sequences FN395039, FR715987, GU071173, EU599094, FN395055, HF586359, FN395056, FR872767, MG029126, FN395031, FN395032, HF586350 and MG029124 were used as reference species for *L. braziliensis, L. panamensis, L. naiffi*, *L. lindenbergi* and *L. infantum* respectively [22, 23, 26–30]. Phylogenetic relationships between *Leishmania* species were inferred by maximum likelihood (ML) with the evolutionary model of Generalized-Time-Reversible *γ* + Invariant (GTR *γ* + I) substitution and 1000 bootstrap replicates using the RAxML-HPC BlackBox software on the CIPRES Science Gateway portal (http://www.phylo.org/). Finally, gene tree in a circular polar form was edited in FigTree [31].

### Genetic diversity and haplotype network

Nucleotide (*π*) and haplotype (*h*) diversity indices were estimated in DNAsp v5.0 [32]. Haplotype network based on *HSP70* for *Leishmania braziliensis* was constructed using the TCS method in Popart software [33] in order to determine nucleotide changes between genetic variants of *Leishmania braziliensis* in Colombia. All sequences of each haplotype were assigned to a specific biogeographical region for Colombia (Additional file 1).

### Statistical analysis

All descriptive and univariate statistical analyzes for patients were developed in IBM SPSS Statistics v25. Non-parametric test was used when the data did not follow Gaussian distribution according to the Kolmogorov-Smirnov test before and after transforming them using base 10 Logarithm. All variables (age, number of lesions, disease evolution, internal and external areas of lesions) were analyzed according to their categorical or quantitative nature and independence of data and were analyzed by the Mann Whitney U test (MWU) and Chi square (X^2^). The variables analyzed in this study were infectious species (*L. braziliensis* and *L. panamensis*), type of treatment (meglumine antimoniate and pentamidine isethionate), internal and external area of lesions, number of lesions, disease evolution (months), age and medical resolution. All tests were two-tailed, and the results were considered significant (*) when *p* < 0.05 and highly significant (**) when *p* < 0.01.

## Results

### Geographical distribution patterns

**Fig. 1:**
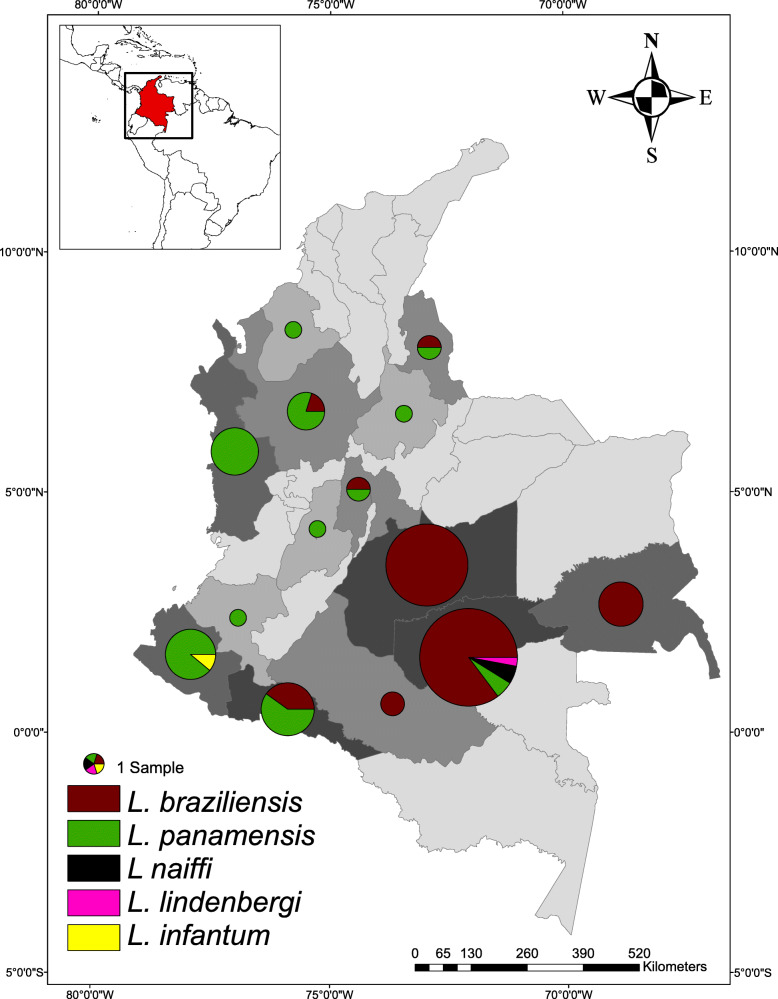
Distribution patterns and relative abundance of *Leishmania* spp. circulating within National Army of Colombia. 65.09% (*n* = 69) of the barcoding identified species corresponded to *L. braziliensis*, while 31.13% (*n* = 33) to *L. panamensis*, only 1.89% (*n* = 2) to *L. naiffi,* 0,945% (*n* = 1) to *L. lindenbergi* and 0.945% (n = 1) to *L. infantum*. Barcoding of the parasite species was carried out by BLASTn with a percentage identity greater than 98% from *HSP70* (106/136) sequences obtained by PCR amplification and terminal dideoxy paired end sequencing for skin smear samples. 106 skin smear samples of patients were analyzed within BONZA, Boyacá (*n* = 74) and BASAN, Bogotá (*n* = 32). (*Own elaboration*)

The 77.94% (106/136) of the patient samples in the study were successfully amplified and sequenced (106/136). The species with the highest relative abundance was *L. braziliensis* with 65.09% (69/106), followed by *L. panamensis* with 31.13% (33/106), *L. naiffi* with 1.89% (2/106), *L. lindenbergi* with 0.945% (1/106) and *L. infantum* with 0.945% (1/106). *L. braziliensis* was reported in eight departments with exclusive distribution in the Amazon, Andean and Orinoco regions, while *L. panamensis* had a wider distribution with report in eleven departments representing four biogeographical regions of Colombia (Amazon, Andean, Caribbean and Pacific) with exception of Insular and Orinoco regions. *L. braziliensis* and *L. panamensis* were identified in reported infected samples from departments such as Antioquia, Cundinamarca, Nariño and Norte de Santander. *L. naiffi* and *L. lindenbergi* were identified by the first time through sequencing for Colombia in the municipality of Miraflores, Guaviare, being the only department with report of four *Leishmania* species in this study. Likewise, *L. infantum* was identified by *HSP70* in the BAS131 patient with CL in the municipality of Tumaco, Nariño department (Fig. 1).

### Phylogenetic relationships

**Fig. 2:**
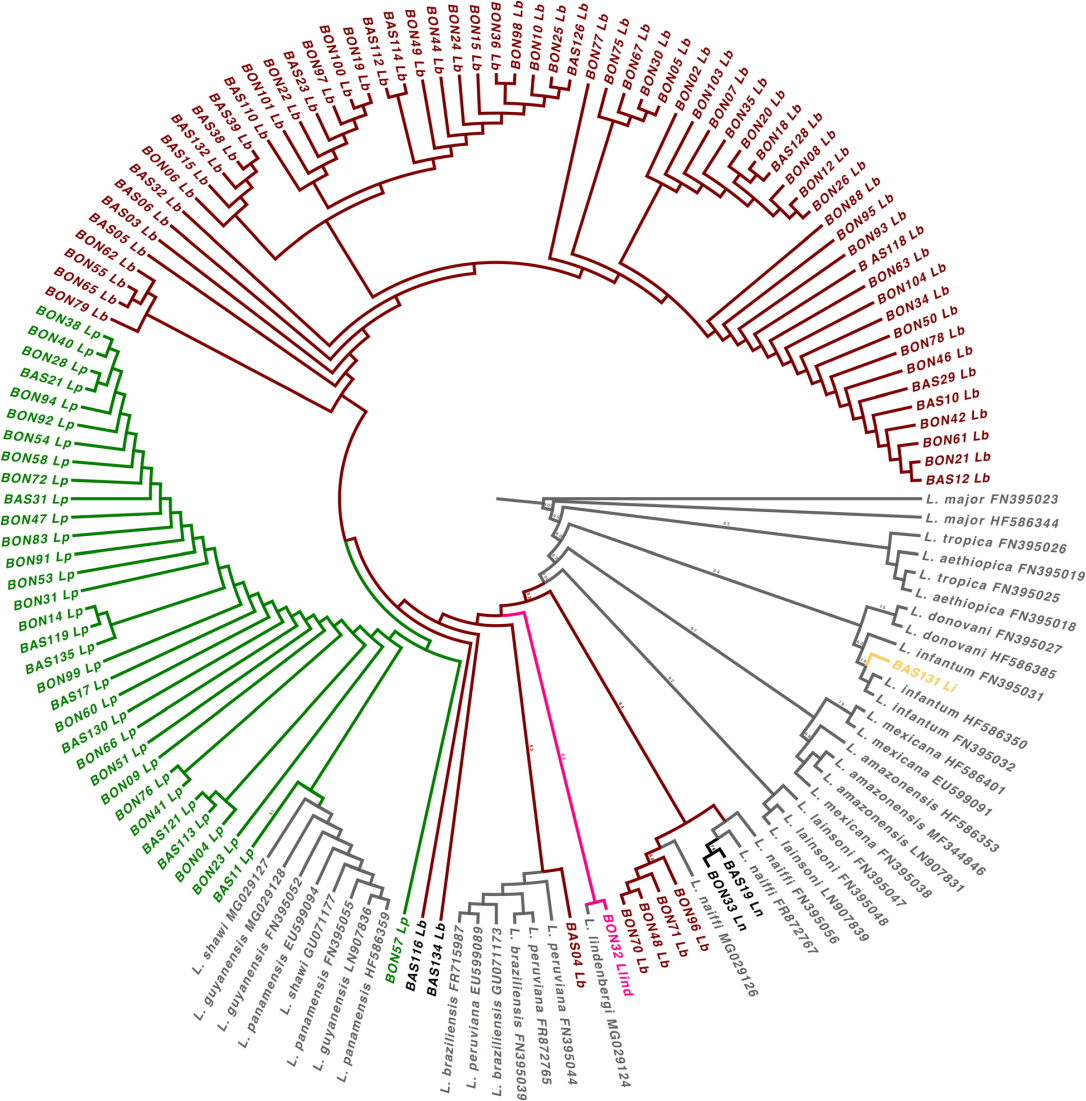
Phylogenetic reconstruction by maximum likelihood of *Leishmania* spp. under GTR *γ* + I evolutionary model according *HSP70* gene. Phylogeny in a circular polar form shows Bootstrap support in branches with percentages of 100% or > 53%. *L. naiffi* is highlighted in black, *L. lindenbergi* in pink, *L. braziliensis* in red wine, *L. panamensis* in dark green and *L. infantum* in yellow. Outgroup: *Leishmania major*. Sister groups: *L. aethiopica*, *L. tropica*, *L donovani*, *L. mexicana*, *L. amazonensis*, *L. lainsoni*, *L. peruviana*, *L. guyanensis* and *L. shawi*. The numbers in the branches tip are sample codes

Gene tree showed phylogenetic consistency for the barcoding confirmation of *Leishmania* species circulating in Colombia (Fig. 2). Bootstrap was reported in branches of main *Leishmania* clades within gene tree based on *HSP70* (Fig. 2). Reciprocal monophyly was defined among all clades, as well as total concordance with NCBI BLASTn barcoding. In the gene tree was found BON32 into *L. lindenbergi* cluster and BON48, BON70, BON71 and BON96 within *L. naiffi* cluster.

### Genetic diversity and haplotype network

**Fig. 3:**
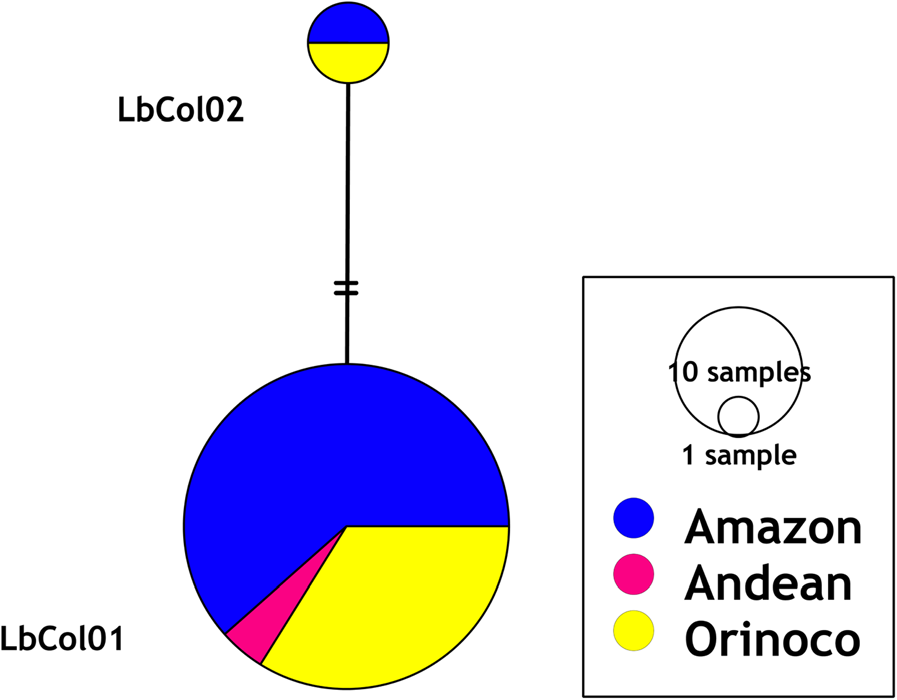
Haplotype network reconstruction for *L. braziliensis* under parsimony criteria with the TCS algorithm according *HSP70* (n = 69, 337 bp). The size of the circles indicates the frequency of each haplotype. Black dot indicates the hypothetical ancestral haplotype and the perpendicular lines between the haplotypes refer to the number of nucleotide substitutions between them

Two haplotypes were found because of two variable sites, a nucleotide diversity *π* = 0.110% (+/− 0.0877%) and a haplotype diversity *h* = 0.111 (+/− 0.0498) for *L. braziliensis* by *HSP70* (NCBI GenBank accession numbers MT543301 and MT543302). No genetic variants were found for *L. panamensis, L. naiffi*, *L. infantum* and *L. lindenbergi* by using *HSP70* (NCBI GenBank accession numbers MT543303 – MT543306). All variable sites for *HSP70* in *L. braziliensis* are described in Additional file 2: Table S1. The genetic diversity indices for *L. naiffi* were not considered due to the low sample size. The haplotype frequency was used in order to do haplotype network according to biogeographical region of possible infection for Colombia (Additional file 2: Table S2). The most frequent haplotype was LbCol01 with greater abundance in the Amazon and Orinoco regions, and present in all eight departments where *L. braziliensis* was notified (Fig. 3).

### Medical resolution, infectious species and clinical-demographic data

The 72.06% (98/136) of the patients were notified with cure according to medical criteria, while 10.29% (14/136) with therapeutic failure and 17.65% (24/136) as without data. This meant an efficacy of the medications during the treatment. Appraising the patients by treatment, 64.29% (63/98) of the patients submitted to the 1st line of meglumine antimoniate had disease healing, while 92.11% (35/38) healed when underwent the 2nd line with pentamidine isethionate (Additional file 3). All patients who had therapeutic failure were referred to a 3rd line treatment according to medical criteria. No significant differences were found in the disease resolution according to the infectious species (*L. braziliensis* versus *L. panamensis*) for meglumine antimoniate (Chi^2^ = 1561; gl = 1), pentamidine isethionate (Chi^2^ = 1105; gl = 1) and both treatments (Chi^2^ = 2.47; gl = 1) (Additional file 3). *L. naiffi* was not considered due to sample size.

Significant differences were found in the medical resolution of lesions (MWU = 381 **) according to patient age, being on average younger those who had therapeutic failure. In contrast, neither lesion areas, nor the disease evolution, nor the number of lesions explained the differences in whether a patient was healed or had a therapeutic failure. However, descriptive statistics such as mean, median and maximum value of the internal and external areas of lesions were higher for those who did not resolve according to medical criteria at the end of treatment (Table 1).

**Table 1:** Descriptive statistics for demographic, clinical and epidemiological data according to medical resolution

	**Age**		**Number of lesions**		**Disease evolution (months)**	
	Healed *n* = 97	TF n = 14	Healed *n* = 98	TF *n* = 14	Healed *n* = 78	TF *n* = 13
MWU	381 (*p* = 0.007) **		635 (*p* = 0.579)		490 (*p* = 0.790)	
**Mean**	24.59	21.79	1.67	1.86	2.73	3.00
**Median**	24.00	20.50	1.00	1.00	2.00	2.00
**Variance**	13.67	7.41	3.58	2.90	5.69	8.00
**Minimum**	19.00	19.00	1.00	1.00	0.04	1.00
**Maximum**	39.00	26.00	13.00	7.00	12.00	10.00
	**Internal area of lesion at the end of treatment**			**External area of lesion at the end of treatment**		
	Healed n = 98	TF n = 12		Healed n = 98	TF *n* = 12	
MWU	575.5 (*p* = 0.646)			506.0 (*p* = 0.260)		
**Mean**	1.02	1.81		2.56	4.29	
**Median**	0.23	0.25		1.00	2.88	
**Variance**	2.34	9.87		14.66	31.89	
**Minimum**	0.00	0.00		0.00	0.00	
**Maximum**	8.70	9.90		24.00	17.50	

MWU refers to the Mann Whitney U test. Patients not analyzed were classified as without data

*TF* Therapeutic Failure

### Clinical history of patients with *L. naiffi*, *L. lindenbergi* and *L. infantum* infection

**Fig. 4:**
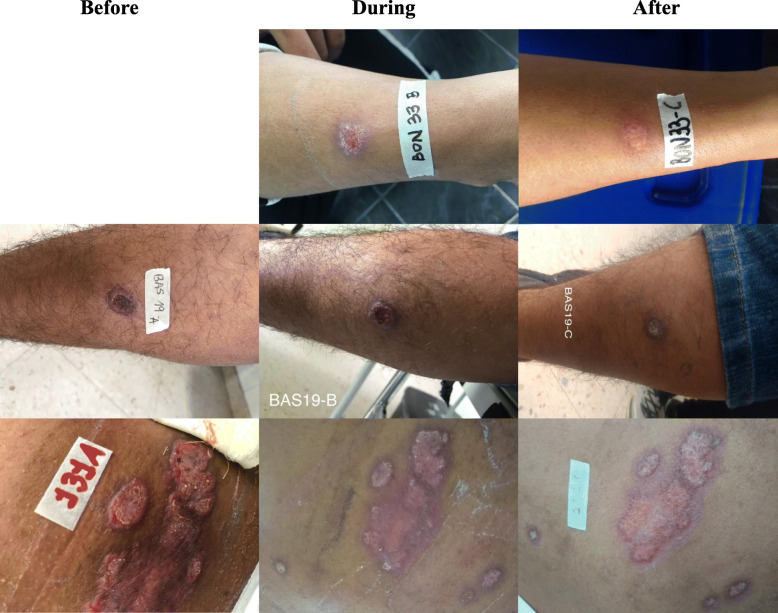
Lesions in patients with CL caused by *L. naiffi* and *L. infantum.* Above is BON33: 19 years old professional soldier with ulcerated lesion on right forearm, possible site of infection in the municipality of Miraflores, Guaviare and with medical resolution as healing after first-line treatment with meglumine antimoniate. In the middle is BAS19: 21 years old regular soldier with ulcerated lesion on right leg, possible site of infection in Miraflores, Guaviare and with medical resolution as healing after first-line treatment with meglumine antimoniate. Below is BAS131: 23 years old professional soldier with plaque-shaped lesion on back, possible site of infection in the municipality of Tumaco, Nariño and with medical resolution as healing at the end of the second-line treatment with pentamidine isethionate

Patient BON33 (*L. naiffi*) and BON32 (*L. lindenbergi*) entered the study on August 15, 2017, being both 19 years old professional soldiers, with 2.5 months of evolution disease before starting first-line treatment with meglumine antimoniate and latter with lesion in left hand. Additionally, patient BAS19 (*L. naiffi*) entered the study on August 3, 2017, as a 21 years old regular soldier, with 3 months of evolution disease before starting first-line treatment with meglumine antimoniate. BON33, BON32 and BAS19 were notified in Miraflores, Guaviare as a potential site of infection. In contrast, the BAS131 (*L. infantum*) patient participated in the study on March 12, 2019 as a 23 years old professional soldier, with therapeutic failure to meglumine antimoniate and before starting second-line treatment with pentamidine isethionate (Fig. 4).

## Discussion

The molecular epidemiology of CL in military population is complex due to continuous movement of troops, internal armed conflict and the sociodemographic features this population has which merits to conduct studies understanding the *Leishmania* species distribution and their potential association with clinical and demographic variables. Herein, we were able to identify several species infecting this vulnerable population (*L. braziliensis, L. panamensis, L. infantum, L. naiffi, L. lindenbergi*). The infectious species with greater relative abundance was *L. braziliensis* followed by *L. panamensis* in accordance with previous reports (Fig. 1) [8, 11]. When comparing our results with Patino et al.*,* 2017, a 5.52% increase in the relative abundance of *L. braziliensis* and a decrease in species richness were observed. The sample size in our study was approximately half compared to the previous study and for Meta department only 25 patients were sampled. Additionally, some differences in relative abundance and richness of species could also be explained by the limitations of inclusion criteria in this study that suggests the need of a more comprehensive sampling to fully unveil the *Leishmania* species distribution in this vulnerable population. In terms of genetic diversity, the indices for *L. braziliensis* according to *HSP70* were much lower in this study compared to indices reported in the military population previously [8] and this could be due to the sampling bias of our study as well.

*Leishmania naiffi* is reported for the first time in Colombia, its main reservoir is the nine-banded armadillo (*Dasypus novemcinctus*) and cutaneous leishmaniasis cases have been reported to date in humans within large areas of the Brazilian Amazon, French Guiana, Ecuador, Peru and Suriname, as well as in Panama and Martinique [34–39]. Its circulation in Colombia was suspected based on *HSP70* through PCR-RFLP [9, 40], consequently, this new report for Colombia highlighted its clinical and epidemiological role in areas where its circulation was not previously known. Another *L. naiffi* reservoir includes animals of rodentia order as Sao Lourenço Punare (*Thrichomys Laurentius*) and Paraguayan punaré (*Thrichomys pachyurus*) endemics for Brazil [41, 42], while the main reported vectors have been *Lutzomyia squamiventris, Lu. paraensis, Lu. davisi, Lu. hirsuta* and Lu. *tortura* in Brazilian and Ecuadorian Amazon as well as *Lu. gomezi* and *Nyssomyia trapidoi* in Panamá [36, 37, 43–46]. In Colombia, *Lu. gomezi, Lu. hirsuta* and *Lu. davisi* have been reported in the Guaviare department [14, 47] explaining a potential transmission cycle for this species in the country. Nevertheless, future studies should consider exploring the transmission cycle of this species and its clinical and biological properties.

*Leishmania lindenbergi* is also reported as a new species circulating in Colombia. To date *L. lindenbergi* is endemic for the Amazon region of Brazil and has been reported as the rarest species with a restricted ecological distribution [48]. Nevertheless, it was reported by the first time in Brazilian soldiers performing activities in secondary forests as well as in a woman from the municipality of Bélem, state of Pará and state of *Rondônia*, Brazil respectively [49]. *Nyssomyia antunesi* has been defined as the main vector playing a determinant role in the transmission cycle of *L. lindenbergi* [49]*.* The patient BON32 reported as the potential place of infection for CL, the municipality of Miraflores, Guaviare. According to previous records of *N. antunesi* for Colombia, this sandfly species was reported in Miraflores, Guaviare as well as mainly in other departments such as Arauca, Caquetá, Casanare, Guajira, Meta, Vaupés and Vichada belonging to Amazon, Caribbean and Orinoco regions of Colombia [14, 50]. In this regard, this would explain a potential transmission scenario for this species in several Colombian regions suggesting the need to conduct new studies to understand the circulating species in unexplored ecotopes for CL, including its vectors.

Regarding the potential association of *Leishmania* species and clinical data, we did not find statistically significant associations. As of today, very few studies have identified a specific *Leishmania* variant with the disease outcome. In our case as most of the samples were *L. braziliensis,* this decreased the statistical power of the test. Then, a more robust epidemiological study must be in place in the light of trying to find evidence associating *Leishmania* species causality and disease outcome. Considering *L. braziliensis* is strongly correlated to MCL in the Americas [51, 52], it was suggested to carry out a clinical and preventive monitoring of patients who were identified with this infectious species and who, according to medical criteria, resulted in therapeutic failure at the end of the study [53]. Similarly, it is important to evaluate in further analyses main factors involved in the therapeutic failure of patients infected with *L. panamensis*. The therapeutic failure may be related to the genetic variants of *Leishmania* species [54]. Moreover, 35 cases of MCL were reported in military personnel between 2018 and 2019 [6] with 51.43% that occurred within departments of Meta (7 cases), Guaviare (6 cases), Santander (3 cases), Putumayo (1 case) and Guainía (1 case), where currently are notified all cases with therapeutic failure according to this study. These departments deserve particular studies regarding virulence and drug resistance by the parasite as well as local transmission cycles evaluating vectors and reservoirs [55, 56]. Further, the presence of *Leishmania RNA virus* (*LRV-1*) in parasites isolated from MCL patients should be explored as this might be related with the virulence of the parasite.

## Conclusions

In conclusion, two new species (*L. naiffi* and *L. lindenbergi*) were reported for the first time in Colombia with possible infection in the municipality of Miraflores, Guaviare. *L. braziliensis* had the greatest relative abundance, while *L. panamensis* had the greatest dispersion within national territory. This confirms previous estimations of these two species circulating in the country. Our findings suggest the need to study unexplored regions as the Amazon and Orinoco where less sampling has been conducted that allowed us to identify these novel species. In addition, we highlight the complex epidemiological scenario of CL in the Colombian military personnel and the need to establish adequate prevention strategies that help to diminish the incidence of CL in this vulnerable population.

## Supplementary Information

**Additional file 1 **Biogeographical regions within Colombia. (*Own elaboration*)

**Additional file 2 **Variable sites and haplotype frequency in *Leishmania* species by possible site of infection.

**Additional file 3.** Demographic, clinical and epidemiological data of patients

## Data Availability

All data sets used or analysed in the current study are included in the supplementary information files of this published article. All genetic sequences derived of current study are deposited and available in GenBank (https://www.ncbi.nlm.nih.gov/genbank/) under accession numbers MT543301, MT543302, MT543303, MT543304, MT543305 and MT543306.

## Abbreviations

CL: Cutaneous leishmaniasis
DNA: Deoxyribonucleic acid
*HSP70*: Heat shock protein 70
MCL: Mucocutaneous leishmaniasis
VBD: Vector borne disease
VL: Visceral leishmaniasis
WHO: World health organization
PAHO: Pan-american health organization
INS: Instituto nacional de salud
SIVIGILA: Sistema nacional de vigilancia en salud pública
BASAN: Batallón de sanidad
CI: Confidence interval
EDTA: Ethylenediamine tetraacetic acid
LRI-DISAN-EJC: Laboratorio de referencia e investigación, dirección de sanidad, ejército
NNN: Novy-macneal-Nicolle medium
cm: Centimeter
μM: Micromolar
μL: Mcrolitre
ng: Nanogram
°C: Degree Celsius
min: Minutes
s: Seconds
bp: Base pairs
TAE: Tris acetate EDTA buffer
MUSCLE: Multiple sequence comparison by log-expectation
BLASTn: Basic local alignment search tool for nucleotide databases using a nucleotide query
NCBI: National Center for Biotechnology Information
ML: Maximum likelihood
GTR *γ* + I: Generalized time reversible gamma and invariant evolutionary model
RAxML-HPC: Randomized axelerated maximum likelihood-high performance computing
CIPRES: Cyberinfrastructure for phylogenetic research
TCS: Templeton-Crandall-Sing algorithm
MWU: Mann whitney u test
BON: Bonza
BAS: BASAN
WD: Without data
PCR-RFLP: Polymerase chain reaction and restriction fragment length polymorphism
TF: Therapeutic failure
NUCL: Non-ulcerative cutaneous leishmaniasis
MLST: Multilocus sequence typing
