# Supplementary figures and images for: Distribution, treatment outcome and genetic diversity of Leishmania species in military personnel from Colombia with cutaneous leishmaniasis
Source: BMC Infect Dis. 2020 Dec 9;20:938. doi: 10.1186/s12879-020-05529-y (PMC7724885; doi:10.1186/s12879-020-05529-y)

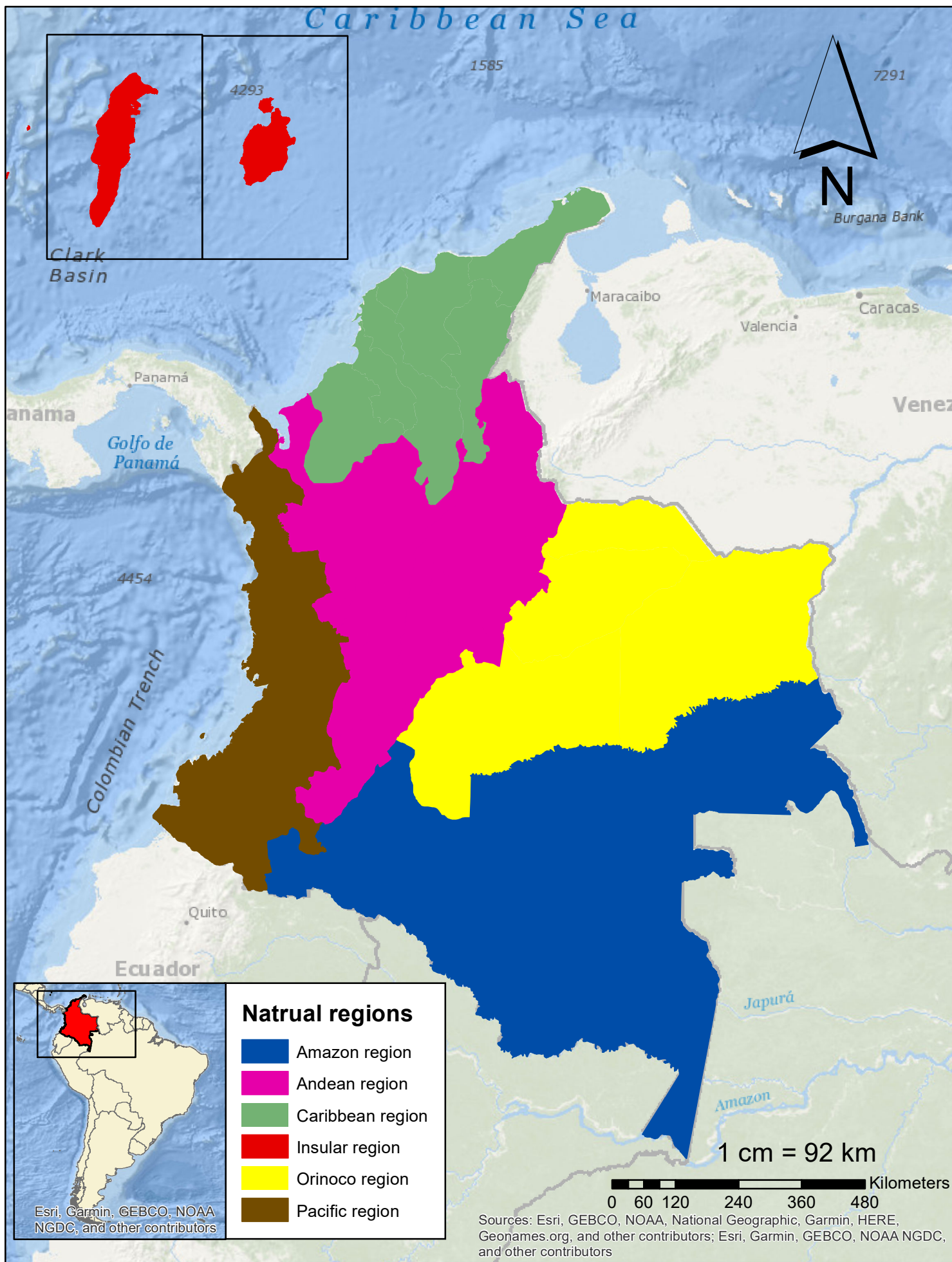

Supplement: Supplementary file 1 — Additional file 1 Biogeographical regions within Colombia. (Own elaboration) [file 12879_2020_5529_MOESM1_ESM.pdf]
