# Supplementary material for: Distribution, treatment outcome and genetic diversity of Leishmania species in military personnel from Colombia with cutaneous leishmaniasis
Source: BMC Infect Dis. 2020 Dec 9;20:938. doi: 10.1186/s12879-020-05529-y (PMC7724885; doi:10.1186/s12879-020-05529-y)
Supplement: Supplementary file 2 — Additional file 2 Variable sites and haplotype frequency in Leishmania species by possible site of infection. [file 12879_2020_5529_MOESM2_ESM.docx]

Additional file 2

Variable sites and haplotype frequency in *Leishmania* species by possible site of infection*.*

**Table S1.** Variable sites in 337bp defining two haplotypes in *L. braziliensis* according to *HSP70* gene.

| *L. braziliensis (HSP70)* | | | |
| --- | --- | --- | --- |
|  | **201** | **210** | **223** |
| **LbCol01** | G | C | G |
| **LbCol02** | A | T | G |

**Table S2.** Haplotype frequency of *Leishmania* species according to *HSP70* (337bp) for the Colombian military population in 106 paired end sequences obtained from skin smear and biopsy sampling between 2017 and 2019.

| **BIOGEOGRAPHICAL REGION** | **AMAZON** | | | | | **ANDEAN** | | | | | **CARIBBEAN** | **ORINOCO** | **PACIFIC** | | |  |  |
| --- | --- | --- | --- | --- | --- | --- | --- | --- | --- | --- | --- | --- | --- | --- | --- | --- | --- |
| **DEPARTMENT** | **CQT** | **GN** | **GVR** | **PTMY** | | **ANTQ** | **CDNMRC** | **NTE STDR** | **STDR** | **TLM** | **CRDB** | **MT** | **CC** | **CHC** | **NRÑ** |  | |
| **GENOTYPES** |  |  |  |  |  | |  |  |  |  |  |  |  |  |  | **TOTAL** | |
| *L. braziliensis (HSP70)* | | | | | | | | | | | | | | | | |  |
| **LbCol01** | 2 | 7 | 28 | 3 | 1 | | 1 | 1 |  |  |  | 22 |  |  |  | **65** | |
| **LbCol02** |  |  | 1 | 1 |  | |  |  |  |  |  | 2 |  |  |  | **4** | |
| **Total** | **2** | **7** | **29** | **4** | **1** | | **1** | **1** |  |  |  | **24** |  |  |  | **n = 69** | |
| *L. panamensis (HSP70)* | | | | | | | | | | | | | | | | |  |
| **LpCol01** |  |  | **2** | **6** | **4** | | **1** | **1** | **1** | **1** | **1** |  | **1** | **7** | **8** | **n = 33** | |
| *L. naiffi* *(HSP70)* | | | | | | | | | | | | | | | | |  |
| **LnCol01** |  |  | **2** |  |  | |  |  |  |  |  |  |  |  |  | **n = 2** | |
| *L. infantum* *(HSP70)* | | | | | | | | | | | | | | | | |  |
| **LiCol01** |  |  |  |  |  | |  |  |  |  |  |  |  |  | **1** | **n = 1** | |
| *L. lindenbergi (HSP70)* | | | | | | | | | | | | | | | | |  |
| **LlindCol01** |  |  | **1** |  |  | |  |  |  |  |  |  |  |  |  | **n = 1** | |

CQT = Caquetá; GN = Guainía; GVR = Guaviare; PTMY = Putumayo; ANTQ = Antioquia; CDNMRC = Cundinamarca; NTE STDR = Norte de Santander; STDR = Santander; TLM = Tolima; CRDB = Córdoba; MT = Meta; CC = Cauca; CHC = Chocó; NRÑ = Nariño.
